# Ultra-early in-cath lab evolocumab achieves rapid lipid target attainment and lipid quality improvement in acute coronary syndrome: a real-world propensity score-matched study

**DOI:** 10.3389/fcvm.2026.1860137

**Published:** 2026-07-22

**Authors:** Yufeng Jiang, Jie Lin, Mingyu Ma, Mei Jiang, Hairong Wang, Jiahong Xu, Pujiao Yu

**Affiliations:** 1 Department of Cardiology, Pudong Gongli Hospital, Shanghai University of Medicine & Health Sciences, Shanghai, China; 2 Department of Neurology, Pudong Gongli Hospital, Shanghai University of Medicine & Health Sciences, Shanghai, China

**Keywords:** acute coronary syndrome, evolocumab, in-cath lab intervention, propensity score matching, small dense low-density lipoprotein

## Abstract

**Background:**

Current guidelines mandate stringent low-density lipoprotein cholesterol (LDL-C) control for patients with acute coronary syndrome (ACS). However, real-world evidence regarding the ultra-early, in-cath lab initiation of PCSK9 inhibitors and their impact on lipid quality—specifically the clearance of highly atherogenic small dense LDL (sdLDL)—remains scarce. This study aimed to evaluate the short-term efficacy and safety of this ultra-early evolocumab intervention strategy.

**Methods:**

This real-world, prospective observational cohort study categorized ACS patients into three treatment groups: statin monotherapy, statin plus ezetimibe, and statin plus evolocumab. Evolocumab was administered ultra-early in the catheterization laboratory immediately after angiographic confirmation. A rigorous propensity score matching (PSM) was conducted to balance baseline characteristics. Primary endpoints included the 4-week LDL-C target achievement rate (<1.4 mmol/L and ≥50% reduction). Secondary endpoints focused on sdLDL clearance and safety outcomes.

**Results:**

From an initial screening pool of 665 patients, a rigorous PSM established a final core analysis cohort of 382 matched patients (127 in the statin monotherapy group, 127 in the ezetimibe group, and 128 in the evolocumab group). At 4 weeks, the evolocumab group demonstrated a substantially higher dual LDL-C target attainment rate compared to the ezetimibe group (66.4% vs. 33.1%, Statin + Evolocumab vs. Statin + Ezetimibe; *P* < 0.001). Additionally, the statin plus ezetimibe group showed significant improvement over statin monotherapy (33.1% vs. 8.7%, Statin + Ezetimibe vs. Statin Monotherapy; *P* < 0.001). Furthermore, evolocumab exhibited a profound superiority in the clearance of pathogenic sdLDL particles, achieving a median reduction rate of 69.08% vs. 48.07% in the ezetimibe group (Statin + Evolocumab vs. Statin + Ezetimibe; *P* < 0.001). Safety profiles were comparable across all groups, with no significant differences in overall adverse events (*P* = 0.171).

**Conclusions:**

The ultra-early, in-cath lab initiation of evolocumab in ACS patients drives rapid, large-scale LDL-C target attainment and demonstrates an absolute advantage in the marked reduction of highly atherogenic sdLDL particles. This deep reversal of the lipid burden provides robust real-world evidence supporting the forward-shifting of intensive lipid-lowering interventions in the acute phase of ACS.

**Trial Registration:**

Chinese Clinical Trial Registry, ChiCTR2600118799.

## Introduction

Acute coronary syndrome (ACS) represents a critical and highly unstable phase of atherosclerotic cardiovascular disease (ASCVD), characterized by a profoundly high risk of recurrent ischemic events, particularly within the first few weeks following the index event (1, 2). Given the well-established causal relationship between cumulative low-density lipoprotein cholesterol (LDL-C) burden and plaque vulnerability (3), rapid and intensive lipid-lowering therapy forms the indisputable cornerstone of secondary prevention. Consequently, current international guidelines, including those from the European Society of Cardiology (ESC) and the European Atherosclerosis Society (EAS), mandate an aggressive dual lipid target for very-high-risk ACS patients: achieving an absolute LDL-C level of < 1.4 mmol/L (55 mg/dL) coupled with a ≥50% reduction from baseline (4).

Despite the widespread prescription of high-intensity statins with or without ezetimibe, a substantial proportion of ACS patients fail to achieve these stringent targets swiftly during the acute phase (5). Traditional oral lipid-lowering agents typically require several weeks to reach their maximal pharmacological effect, leaving patients exposed to a dangerous “vulnerability window” (6). Proprotein convertase subtilisin/kexin type 9 (PCSK9) inhibitors, such as evolocumab, have demonstrated unprecedented efficacy in drastically lowering LDL-C levels and improving cardiovascular outcomes (7, 8). However, the optimal timing for their initiation remains a subject of ongoing debate. While recent trials have explored early administration during hospitalization (9, 10), real-world evidence evaluating an “ultra-early” strategy—specifically, initiating evolocumab directly in the catheterization laboratory (in-cath lab) immediately following angiographic confirmation—is exceedingly scarce.

Beyond absolute quantitative reductions in LDL-C, qualitative alterations in lipoproteins are increasingly recognized as pivotal drivers of plaque instability in the acute phase of ACS (11). Among these, small dense LDL (sdLDL) particles are notoriously atherogenic due to their prolonged circulation time, enhanced susceptibility to oxidation, and greater penetration into the arterial intima (12, 13). Although previous research, such as the landmark study by Sliz et al. (14) has elegantly demonstrated that PCSK9 inhibition significantly alters lipoprotein subclass profiles and improves overall ‘lipid quality’ beyond standard LDL-C reductions, its specific impact in the acute emergency setting remains a critical knowledge gap. The precise magnitude and speed of sdLDL clearance following ultra-early evolocumab administration during ad-hoc PCI for ACS is still largely unexplored.

In light of these critical knowledge gaps, we hypothesized that the ultra-early, in-cath lab initiation of evolocumab would rapidly bridge the treatment gap by driving swift LDL-C target attainment and profound sdLDL clearance without compromising safety. Therefore, this real-world, propensity score-matched (PSM) study was designed to rigorously evaluate the short-term efficacy, lipid quality improvement, and safety profile of an in-cath lab evolocumab intervention strategy compared to standard care in patients presenting with ACS.

## Methods

### Study design and setting

This study was a real-world, prospective, observational cohort study incorporating a rigorous propensity score-matching (PSM) analysis. The cornerstone of our study design was the implementation of an “in-cath lab ultra-early intervention strategy.” All enrolled patients completed the intervention during the perioperative period of percutaneous coronary intervention (PCI) immediately after being diagnosed with acute coronary syndrome (ACS) in the emergency setting. The study was conducted at Shanghai Pudong New Area Gongli Hospital between January 2024 and December 2025, with a predefined short-term clinical and laboratory follow-up period of 4 weeks.

### Ethical approval and consent

The study protocol was conducted in strict adherence to the ethical principles outlined in the Declaration of Helsinki (15) and was approved by the Institutional Review Board and Ethics Committee of Shanghai Pudong New Area Gongli Hospital (Approval No.: GLYY1s-055). Furthermore, the study protocol was registered with the Chinese Clinical Trial Registry (Identifier: ChiCTR2600118799). Given the critical and time-sensitive nature of emergency PCI for ACS, where minimizing door-to-balloon time is paramount, an ethically approved “deferred consent” (waiver of prior informed consent in emergency research) procedure was implemented (16). During the acute resuscitation and pre-operative phase, brief verbal assent was obtained from the patients or accompanying relatives. Comprehensive written informed consent was subsequently secured from the patients or their legally authorized representatives once the patients were clinically stable, typically within 24 h post-procedure.

### Study population

The study prospectively screened consecutive ACS patients presenting to the emergency department. The inclusion criteria were defined as follows: (1) age ≥18 years; (2) a confirmed primary diagnosis of ACS, encompassing ST-segment elevation myocardial infarction (STEMI), non-ST-segment elevation myocardial infarction (NSTEMI), and unstable angina (UA) (17); and (3) angiographically confirmed atherosclerotic cardiovascular disease (ASCVD) where the cardiovascular team made an immediate decision to perform ad-hoc PCI.

To strictly control for inflammatory confounders and isolate plaque-driven mechanisms, the exclusion criteria were rigorously established as: (1) angiographic evidence suggesting non-atherosclerotic etiologies, such as coronary artery spasm, coronary microvascular dysfunction, or myocardial infarction with non-obstructive coronary arteries (MINOCA) (18); (2) patients scheduled for coronary artery bypass grafting (CABG) or requiring solely conservative medical management following angiography; (3) any prior exposure to PCSK9 inhibitors; (4) concomitant severe active infections, autoimmune diseases, or chronic inflammatory conditions [to preclude non-cardiovascular interference in the evaluation of high-sensitivity C-reactive protein (hs-CRP)]; (5) severe hepatic or renal impairment (e.g., estimated glomerular filtration rate < 30 mL/min/1.73m^2^); and (6) known contraindications or a history of severe hypersensitivity to the study medications (statins, ezetimibe, or PCSK9 inhibitors).

### Treatment protocols and intervention timing

To reflect real-world clinical practice in the highly time-sensitive setting of emergency percutaneous coronary intervention (PCI), this study was designed as a prospective observational cohort. Rather than utilizing strict randomization—which is often constrained outside of highly controlled trial environments—treatment assignments were guided by routine clinical workflows, specifically the attending interventional cardiologist's immediate judgment and shared decision-making at the bedside. Recognizing that this pragmatic approach inherently introduces allocation bias, a rigorous Propensity Score Matching (PSM) analysis was subsequently implemented to robustly adjust for confounding variables. This stratifies patients into three highly comparable treatment arms: the statin monotherapy group, the statin plus ezetimibe group, and the statin plus evolocumab group. For the evolocumab group, an “ultra-early” administration protocol was strictly enforced: immediately following angiographic confirmation of the culprit lesion and prior to the passage of the PCI guidewire, an initial subcutaneous injection of evolocumab (140 mg) was administered directly in the catheterization laboratory. Following this index procedure, patients continued a standard maintenance regimen, receiving a subsequent 140 mg injection of evolocumab exactly 14 days later (Q2W) during the 4-week follow-up period. This was complemented by background moderate-to-high-intensity statin therapy (e.g., atorvastatin 20 mg/day or rosuvastatin 10 mg/day). In the active control arms, ezetimibe (10 mg/day) plus a statin, or statin monotherapy alone, was initiated during the very early phase (within 24 h of symptom onset or admission) according to standard clinical pathways. All patients concomitantly received guideline-directed medical therapy for ACS, including dual antiplatelet therapy and beta-blockers, ensuring strict consistency in baseline cardiovascular care (19).

### Blood sampling and laboratory analysis

To precisely capture the acute stress-induced lipid and inflammatory burden while minimizing patient discomfort during emergency PCI, a specialized arterial sheath blood sampling protocol was utilized. Baseline (T0) blood samples were drawn directly from the arterial sheath immediately after the completion of diagnostic angiography and systemic heparinization, prior to the PCI intervention. Recognizing the non-fasting state of these emergency presentations—a condition now widely endorsed by the European Atherosclerosis Society (EAS) for clinical risk assessment (20)—we rigorously eschewed the traditional Friedewald formula (21). Instead, to eliminate non-fasting interference, baseline and 4-week follow-up lipid profiles—specifically LDL-C and sdLDL levels—were directly quantified using automated homogeneous enzymatic colorimetric assays (utilizing selective surface-active detergents to isolate specific lipoprotein fractions) on an AU5800 automated chemistry analyzer (Beckman Coulter, Brea, CA, USA). This direct measurement method completely bypassed the need for the traditional Friedewald calculation, ensuring robust analytical accuracy and reproducibility irrespective of the patient's acute dietary or fasting state. Follow-up laboratory assessments were conducted at 4 weeks using standard fasting venous blood samples (following an 8-to-12-hour fast) during outpatient visits.

### Study endpoints and statistical analysis

The primary efficacy endpoint was the dual target achievement rate for LDL-C at the 4-week follow-up, stringently defined as attaining an absolute LDL-C level <1.4 mmol/L (55 mg/dL) coupled with a ≥50% reduction from baseline. Secondary and mechanistic endpoints encompassed the absolute and percentage clearance of highly atherogenic sdLDL particles. Safety endpoints included the incidence of statin-associated muscle symptoms, hepatic enzyme elevations (ALT or AST >3× upper limit of normal), and injection-site reactions. Injection-site reactions were strictly defined as local adverse events (e.g., erythema, pain, or swelling) directly associated with the subcutaneous administration of evolocumab. Localized bruising resulting from routine venipuncture for blood sampling was rigorously excluded from this specific category.

To rigorously mitigate the inherent confounding biases of observational designs, a propensity score-matching (PSM) analysis was performed (22). The propensity score was estimated using a multivariable logistic regression model incorporating key clinical covariates: age, gender, body mass index, smoking status, medical history (hypertension, diabetes, prior MI), and baseline lipid parameters. Matching was executed using the nearest-neighbor algorithm with a strict caliper width of 0.2 (23). The balance of covariates post-matching was evaluated using the standardized mean difference (SMD).

Continuous variables are presented as mean ± standard deviation (SD) or median [interquartile range (IQR)], and categorical variables as frequencies (percentages). Baseline characteristics were compared using one-way analysis of variance (ANOVA), the Kruskal–Wallis test, or the Chi-square test, as appropriate, with Tukey's or Mann–Whitney U tests for *post-hoc* comparisons. To evaluate 4-week continuous lipid measurements and their absolute changes, Analysis of Covariance (ANCOVA) was employed, adjusting for respective baseline values. To satisfy normality assumptions, skewed variables (e.g., sdLDL, triglycerides) were natural log-transformed prior to ANCOVA. Percentage changes from baseline were assessed using the Kruskal–Wallis test, followed by Mann–Whitney U tests for pairwise comparisons. Subgroup analyses for the primary endpoint compared the evolocumab and statin plus ezetimibe arms. Interaction effects (P for interaction) were evaluated by incorporating a two-way interaction term (treatment arm  ×  subgroup variable) into the multivariable logistic regression models. Analyses were conducted using SPSS 26.0 and R software. A two-sided *P* < 0.05 indicated statistical significance.

## Results

### Study population and baseline characteristics

**Figure 1:**
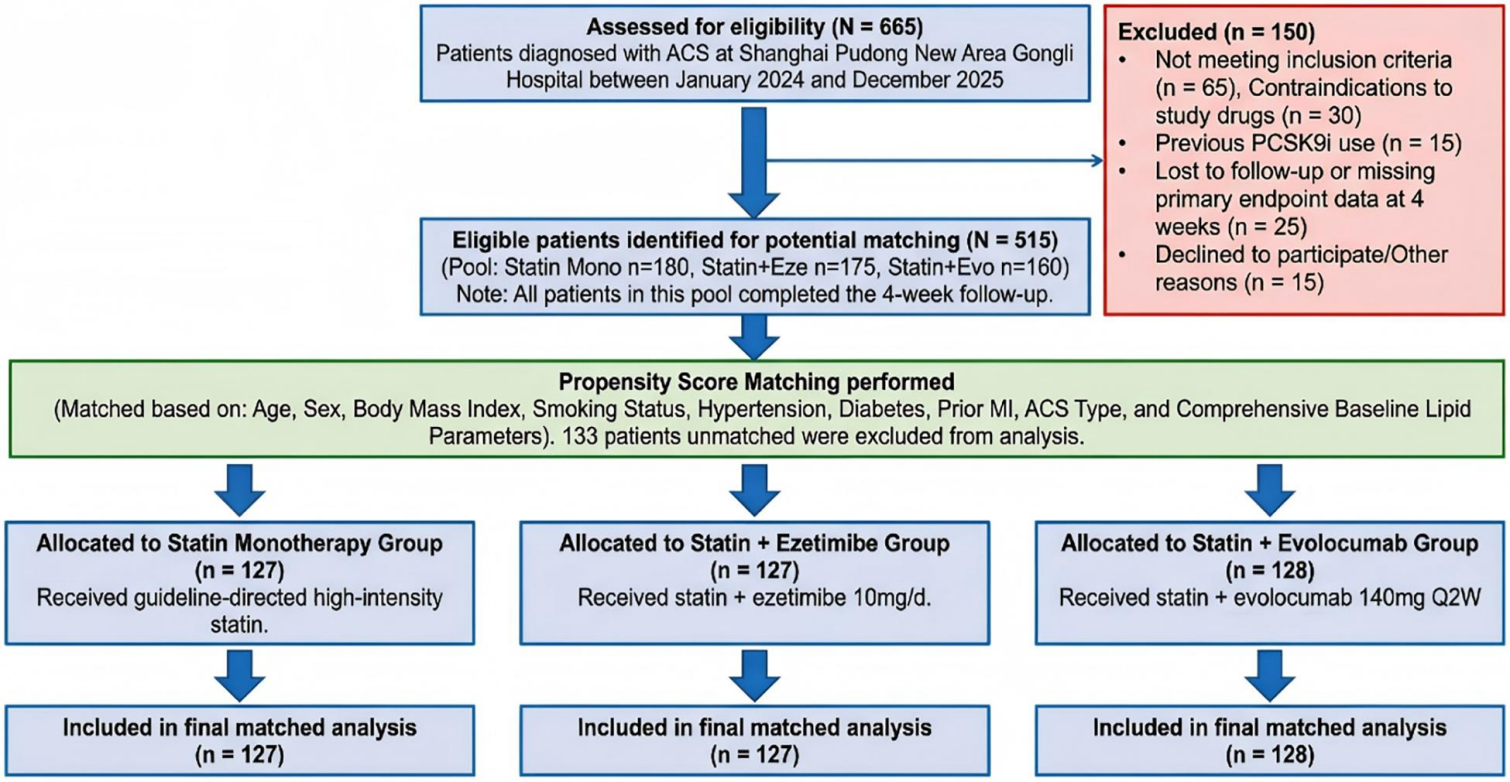
Study flowchart and patient selection process. A total of 665 patients diagnosed with acute coronary syndrome (ACS) between January 2024 and December 2025 were initially screened. After excluding 150 patients based on predefined criteria, 515 eligible patients entered the pool for propensity score matching. Ultimately, 133 unmatched patients were excluded, and 382 patients were successfully matched into three groups: Statin Monotherapy (*n* = 127), Statin + Ezetimibe (*n* = 127), and Statin + Evolocumab (*n* = 128).

Between January 2024 and December 2025, a total of 665 patients diagnosed with ACS were initially assessed for eligibility at our institution. After excluding 150 patients based on the predefined criteria (such as not meeting inclusion criteria, presence of contraindications, or prior PCSK9i use), 515 eligible patients entered the pre-matching cohort. To mitigate allocation bias, a rigorous propensity score matching (PSM) was performed. Consequently, 133 unmatched patients were excluded, establishing a final core analysis cohort of 382 perfectly matched patients (127 in the statin monotherapy group, 127 in the statin plus ezetimibe group, and 128 in the evolocumab group; Figure 1 and Table 1). Importantly, in strict adherence to the study protocol, all 382 matched patients (100%) successfully underwent ad-hoc percutaneous coronary intervention (PCI) for the culprit lesions immediately following diagnostic angiography. As detailed in Table 1, the matching process achieved a high degree of balance across baseline demographics, including age and body mass index (BMI). Notably, the core baseline lipid parameters were exceptionally well-balanced among the three groups, including baseline LDL-C levels (statin monotherapy: 3.60 ± 0.92 mmol/L; statin plus ezetimibe: 3.45 ± 0.76 mmol/L; statin plus evolocumab: 3.49 ± 0.86 mmol/L; *P* = 0.369) and baseline sdLDL concentrations (*P* = 0.258). Although a marginal statistical difference was observed in the overall comparison of baseline triglyceride (TG) levels (*P* = 0.019), the standardized mean difference (SMD) between the core evolocumab and ezetimibe groups was only 0.177 (<0.20). This confirms that a high degree of clinical balance was successfully achieved between the primary comparison arms, demonstrating that the overall P-value was largely driven by sample size effects rather than true clinical confounding. Overall, PSM successfully mitigated the confounding biases inherent in non-randomized observational allocations, establishing a robustly equitable foundation for subsequent efficacy comparisons.

**Table 1:** Baseline demographic and clinical characteristics of the study population after propensity score matching.

Variable	Statin Monotherapy (*n* = 127)	Statin + Ezetimibe (*n* = 127)	Statin + Evolocumab (*n* = 128)	P-value
N	127	127	128	-
Age (years)	60.85 ± 10.40	61.79 ± 10.18	61.58 ± 10.14	0.746
BMI (kg/m^2^)	25.40 ± 2.96	25.24 ± 3.08	25.68 ± 3.24	0.519
LDL-C (mmol/L)	3.60 ± 0.92	3.45 ± 0.76	3.49 ± 0.86	0.369
HDL-C (mmol/L)	1.09 ± 0.23	1.11 ± 0.25	1.10 ± 0.24	0.921
TG (mmol/L)	1.72 ± 0.69	1.50 ± 0.54	1.60 ± 0.59	0.019
TC (mmol/L)	5.52 ± 1.01	5.28 ± 0.83	5.36 ± 0.97	0.123
non-HDL (mmol/L)	4.42 ± 1.01	4.17 ± 0.81	4.26 ± 0.94	0.092
ApoB (g/L)	0.92 ± 0.22	0.90 ± 0.20	0.89 ± 0.21	0.456
ApoA1 (g/L)	1.26 ± 0.27	1.27 ± 0.30	1.27 ± 0.28	0.868
sdLDL (mg/L)	332.20 [282.70–397.80]	325.90 [270.45–372.00]	326.80 [280.75–375.67]	0.258
Lpa (mg/L)	193.90 [120.45–307.60]	191.00 [124.50–292.50]	170.85 [114.22–269.33]	0.526
CRP (mg/L)	17.68 [10.55–29.90]	16.70 [9.94–31.59]	22.41 [10.58–40.98]	0.16
TNI (ng/L)*	9.60 [1.95–18.42]	9.64 [3.00–15.75]	9.07 [0.04–16.30]	0.709
BNP (pg/mL)*	260.90 [172.00–463.80]	247.90 [140.80–516.45]	239.90 [141.20–445.15]	0.643
Gender				
Male	82 (64.6%)	87 (68.5%)	79 (61.7%)	0.522
Female	45 (35.4%)	40 (31.5%)	49 (38.3%)	
Smoking				
Yes	53 (41.7%)	47 (37.0%)	61 (47.7%)	0.226
No	74 (58.3%)	80 (63.0%)	67 (52.3%)	
Hypertension				
No	40 (31.5%)	50 (39.4%)	46 (35.9%)	0.422
Yes	87 (68.5%)	77 (60.6%)	82 (64.1%)	
Diabetes				
Yes	46 (36.2%)	51 (40.2%)	45 (35.2%)	0.685
No	81 (63.8%)	76 (59.8%)	83 (64.8%)	
Prior MI				
Yes	15 (11.8%)	21 (16.5%)	15 (11.7%)	0.434
No	112 (88.2%)	106 (83.5%)	113 (88.3%)	
ACS Type				
STEMI	45 (35.4%)	46 (36.2%)	46 (35.9%)	0.431
UA	26 (20.5%)	25 (19.7%)	36 (28.1%)	
NSTEMI	56 (44.1%)	56 (44.1%)	46 (35.9%)	

Data are presented as mean ± standard deviation (SD) for normally distributed continuous variables, median [interquartile range, Q1–Q3] for skewed continuous variables, or number (percentage) for categorical variables. The P-value indicates the overall comparison among the three matched groups.

BMI, body mass index; LDL-C, low-density lipoprotein cholesterol; HDL-C, high-density lipoprotein cholesterol; TG, triglycerides; TC, total cholesterol; ApoB, apolipoprotein B; ApoA1, apolipoprotein A1; sdLDL, small dense low-density lipoprotein; Lpa, lipoprotein(a); CRP, C-reactive protein; TNI, troponin I; BNP, B-type natriuretic peptide; MI, myocardial infarction; ACS, acute coronary syndrome; STEMI, ST-segment elevation myocardial infarction; NSTEMI, non-ST-segment elevation myocardial infarction; UA, unstable angina.

### Primary efficacy: rapid LDL-C target attainment

**Figure 2:**
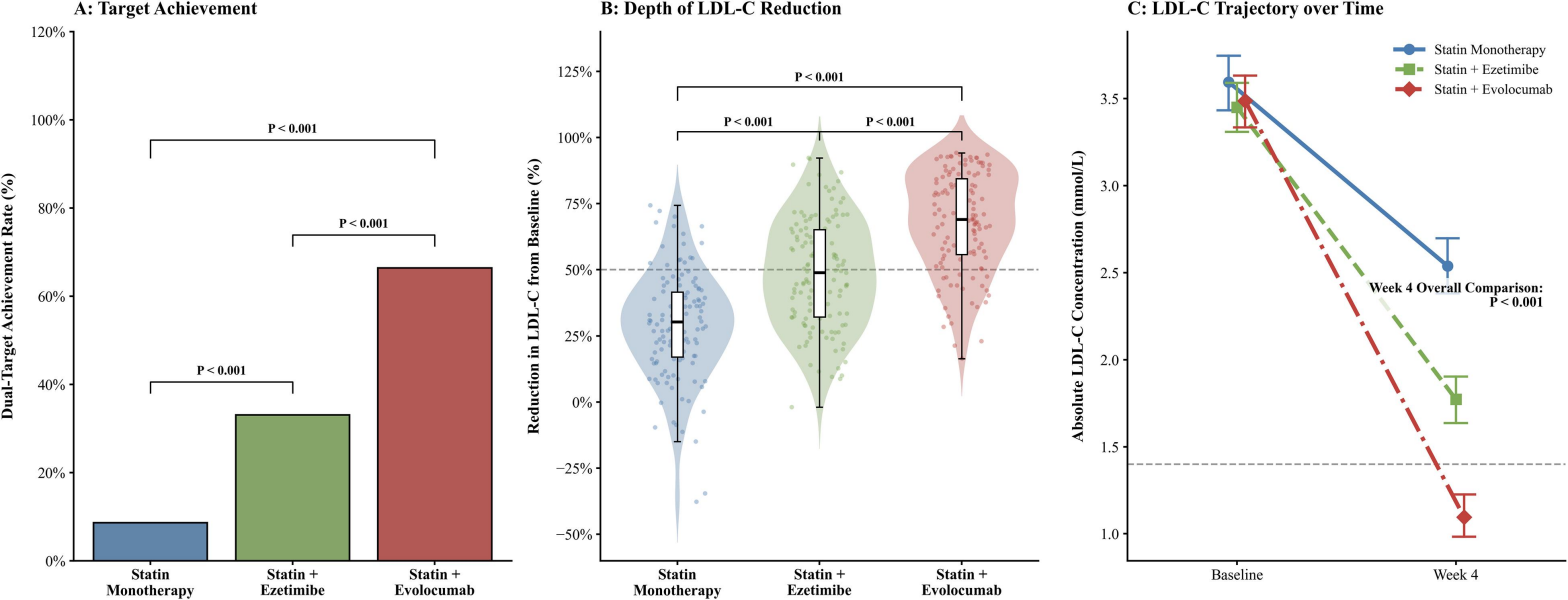
Efficacy outcomes: strict dual LDL,C target achievement,depth of reduction,and trajectory at the 4-week folloW,Up. **(A)** Target Achievement Rate: The bar chart compares the percentage of patients achieving the stringent dual target (defined as an absolute LDL-C level <1.4 mmol/L AND a ≥ 50% reduction from baseline) across the three cohorts. **(B)** Depth of LDL,C Reduction: Violin and superimposed box plots illustrating the profound percentage reduction rates of LDL-C from baseline. The dashed red line indicates the 50% reduction target. **(C)** LDL,C Trajectory over Time: Point plots tracking the absolute LDL-C concentration from baseline to 4 weeks, with the dashed red line representing the 1.4 mmol/L absolute target. Crucially, P-values for all pairwise comparisons are displayed across the panels, illustrating a statistically significant stepwise escalation in efficacy from statin monotherapy to statin plus ezetimibe, and a profound further improvement with the ultra-early addition of evolocumab. P-values were calculated using the Chi-square test (Panel A), the Kruskal–Wallis and Mann–Whitney U tests (Panel B), and Analysis of Covariance (ANCOVA) (Panel C overall comparison), where appropriate.

At the 4-week follow-up, the ultra-early evolocumab intervention demonstrated an overwhelming lipid-lowering superiority (Table 2). As expected, the addition of ezetimibe to background statin therapy yielded a statistically significant, stepwise incremental benefit over statin monotherapy alone (dual LDL-C target attainment rate: 33.1% vs. 8.7%, Statin + Ezetimibe vs. Statin Monotherapy; *P* < 0.001; absolute LDL-C reduction: 1.68 ± 0.81 vs. 1.06 ± 0.79 mmol/L, Statin + Ezetimibe vs. Statin Monotherapy; *P* < 0.001). However, the most striking finding remains the exponential therapeutic leap demonstrated by the ultra-early, in-cath lab initiation of evolocumab, which achieved a profound absolute LDL-C decrease of 2.39 ± 0.93 mmol/L (Statin + Evolocumab vs. Statin + Ezetimibe; *P* < 0.001). More importantly, as illustrated in Figure 2, regarding the highly stringent primary clinical endpoint of “dual target achievement” (defined as an absolute LDL-C < 1.4 mmol/L coupled with a ≥ 50% reduction from baseline), the evolocumab group attained a substantial rate of 66.4% (85/128), which was nearly double that of the ezetimibe group (33.1%, 42/127; Statin + Evolocumab vs. Statin + Ezetimibe; *P* < 0.001) and vastly outpaced standard monotherapy. These findings unequivocally validate the immense efficacy of the in-cath lab ultra-early PCSK9 inhibitor administration in driving rapid lipid target attainment.

**Table 2:** Efficacy outcomes and dynamic changes in lipid profiles at the 4-week follow-up.

Efficacy Outcome	Statin Monotherapy (*n* = 127)	Statin + Ezetimibe (*n* = 127)	Statin + Evolocumab (*n* = 128)	Overall P-value	P-value (Evo vs. Eze)	P-value (Eze vs. Mono)
Target Achievement						
LDL-C < 1.4 mmol/L	12 (9.4%)	44 (34.6%)	88 (68.8%)	<0.001	<0.001	<0.001
LDL-C Reduction >= 50%	17 (13.4%)	59 (46.5%)	105 (82.0%)	<0.001	<0.001	<0.001
Both Targets Achieved	11 (8.7%)	42 (33.1%)	85 (66.4%)	<0.001	<0.001	<0.001
LDL-C (mmol/L)						
Baseline	3.60 ± 0.92	3.45 ± 0.76	3.49 ± 0.86	0.369	0.717	0.158
4-Week Follow-up	2.54 ± 0.96	1.77 ± 0.80	1.10 ± 0.71	<0.001	<0.001	<0.001
Absolute Reduction	1.06 ± 0.79	1.68 ± 0.81	2.39 ± 0.93	<0.001	<0.001	<0.001
Reduction Rate (%)	29.04 ± 20.28	48.50 ± 20.67	68.04 ± 18.79	<0.001	<0.001	<0.001
TG (mmol/L)						
Baseline	1.72 ± 0.69	1.50 ± 0.54	1.60 ± 0.59	0.019	0.166	0.005
4-Week Follow-up	1.58 ± 0.66	1.37 ± 0.50	1.47 ± 0.55	0.018	0.129	0.005
Absolute Reduction	0.15 ± 0.18	0.14 ± 0.14	0.13 ± 0.14	0.776	0.886	0.622
Reduction Rate (%)	8.38 ± 8.80	8.71 ± 7.54	8.29 ± 7.87	0.91	0.666	0.749
TC (mmol/L)						
Baseline	5.52 ± 1.01	5.28 ± 0.83	5.36 ± 0.97	0.123	0.47	0.04
4-Week Follow-up	4.42 ± 1.01	3.60 ± 0.89	2.96 ± 0.86	<0.001	<0.001	<0.001
Absolute Reduction	1.10 ± 0.79	1.68 ± 0.80	2.40 ± 0.94	<0.001	<0.001	<0.001
Reduction Rate (%)	19.53 ± 13.10	31.65 ± 13.86	44.31 ± 14.04	<0.001	<0.001	<0.001
sdLDL (mg/L)						
Baseline	332.20 [282.70–397.80]	325.90 [270.45–372.00]	326.80 [280.75–375.67]	0.258	0.637	0.165
4-Week Follow-up	227.90 [183.95–290.95]	162.50 [110.25–216.55]	102.95 [52.77–144.92]	<0.001	<0.001	<0.001
Absolute Reduction	95.20 [53.40–141.50]	147.60 [104.05–209.15]	211.95 [161.32–285.82]	<0.001	<0.001	<0.001
Reduction Rate (%)	30.60 [17.16–40.65]	48.07 [34.03–64.08]	69.08 [53.38–83.67]	<0.001	<0.001	<0.001
HDL-C (mmol/L)						
Baseline	1.09 ± 0.23	1.11 ± 0.25	1.10 ± 0.24	0.921	0.955	0.508
4-Week Follow-up	1.13 ± 0.25	1.15 ± 0.26	1.15 ± 0.26	0.66	0.992	0.533
Absolute Change	0.03 ± 0.06	0.05 ± 0.06	0.05 ± 0.06	0.06	0.84	0.01
Percent Change (%)	3.00 ± 5.34	4.34 ± 5.06	4.24 ± 4.86	0.065	0.884	0.041
non-HDL (mmol/L)						
Baseline	4.42 ± 1.01	4.17 ± 0.81	4.26 ± 0.94	0.092	0.445	0.03
4-Week Follow-up	3.29 ± 1.02	2.44 ± 0.85	1.81 ± 0.77	<0.001	<0.001	<0.001
Absolute Reduction	1.13 ± 0.79	1.73 ± 0.80	2.45 ± 0.94	<0.001	<0.001	<0.001
Reduction Rate (%)	25.23 ± 16.51	41.32 ± 17.29	57.03 ± 16.43	<0.001	<0.001	<0.001
ApoA1 (g/L)						
Baseline	1.26 ± 0.27	1.27 ± 0.30	1.27 ± 0.28	0.868	0.902	0.78
4-Week Follow-up	1.30 ± 0.29	1.33 ± 0.30	1.33 ± 0.30	0.558	0.954	0.413
Absolute Change	0.04 ± 0.09	0.06 ± 0.09	0.06 ± 0.08	0.111	0.527	0.075
Percent Change (%)	3.47 ± 7.39	4.75 ± 6.79	5.06 ± 6.52	0.149	0.707	0.152
ApoB (g/L)						
Baseline	0.92 ± 0.22	0.90 ± 0.20	0.89 ± 0.21	0.456	0.624	0.449
4-Week Follow-up	0.70 ± 0.24	0.55 ± 0.21	0.40 ± 0.18	<0.001	<0.001	<0.001
Absolute Reduction	0.22 ± 0.20	0.36 ± 0.20	0.49 ± 0.25	<0.001	<0.001	<0.001
Reduction Rate (%)	23.60 ± 19.97	38.85 ± 19.58	53.65 ± 20.98	<0.001	<0.001	<0.001

Data are unadjusted descriptive statistics [mean ± SD, or median (Q1–Q3) for skewed variables like sdLDL] to reflect raw clinical distributions. Dual target: absolute LDL-C < 1.4 mmol/L and ≥50% reduction. Overall P-values compare all three groups; pairwise P-values compare Statin + Ezetimibe vs. Monotherapy (Eze vs. Mono), and Statin + Evolocumab vs. Statin + Ezetimibe (Evo vs. Eze). Baseline comparisons utilized ANOVA, Kruskal–Wallis, or Chi-square tests. For 4-week measurements and absolute changes, reported P-values were derived from ANCOVA (adjusted for baseline covariates); severely skewed variables (e.g., sdLDL, TG) were log-transformed prior to ANCOVA to satisfy normality. Percent changes were evaluated using Kruskal–Wallis and Mann–Whitney U tests. LDL-C, low-density lipoprotein cholesterol; TG, triglycerides; TC, total cholesterol; sdLDL, small dense low-density lipoprotein; HDL-C, high-density lipoprotein cholesterol; non-HDL, non-high-density lipoprotein cholesterol; ApoA1, apolipoprotein A1; ApoB, apolipoprotein B.

### Secondary efficacy: improvement in lipid quality and sdLDL clearance

**Figure 3:**
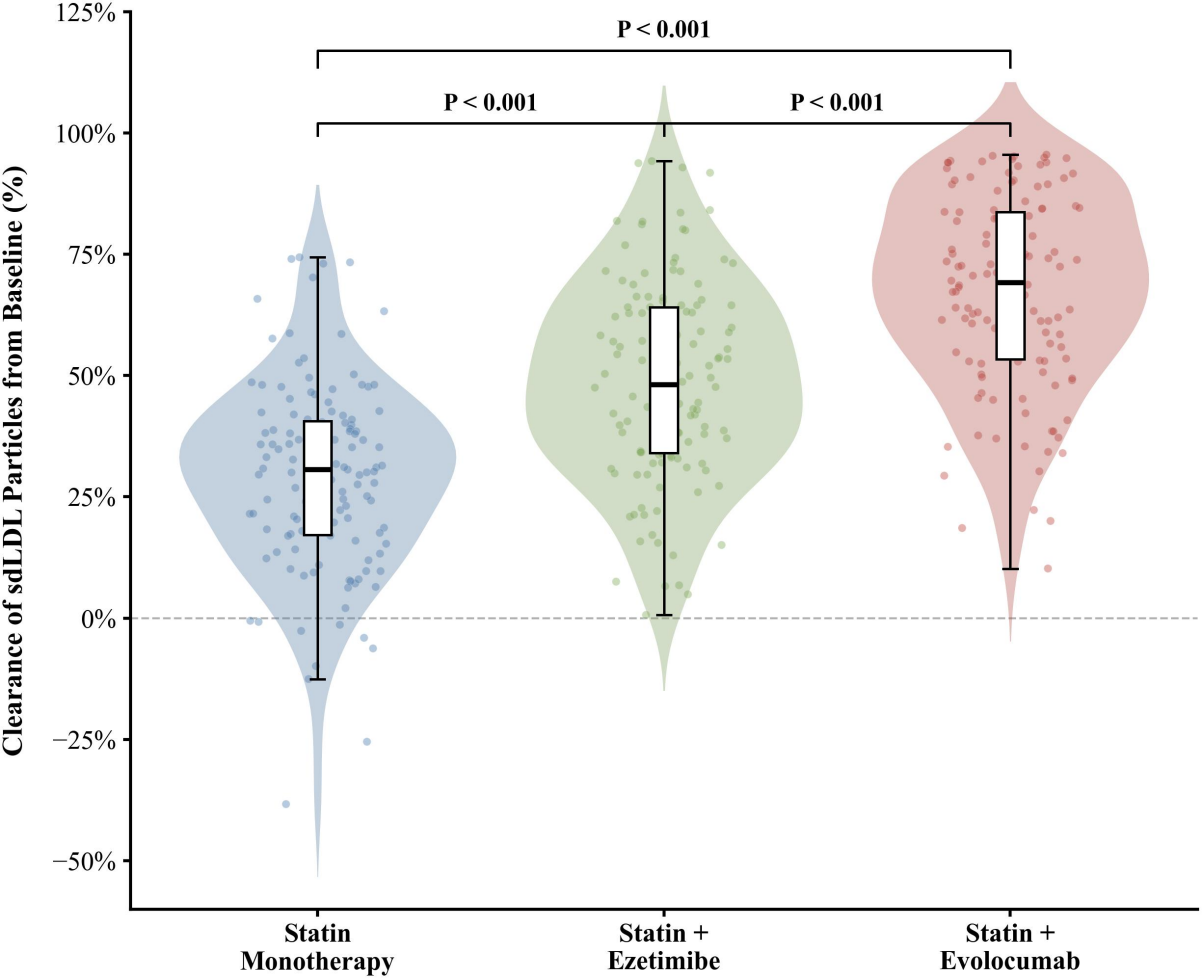
Depth of highly atherogenic small dense loW, density lipoprotein (sdLDL) clearance at 4 weeks. The violin plots, superimposed box plots, and jittered scatter points illustrate the profound percentage reduction rates of highly atherogenic sdLDL particles from baseline. The Statin + Evolocumab group demonstrated a drastic downward shift in sdLDL distribution, achieving the highest percentage clearance compared to standard oral therapies, indicating a significant improvement in overall lipid quality. Crucially, P-values for all pairwise comparisons are displayed across the groups, demonstrating a significant stepwise enhancement in sdLDL clearance. Pairwise P-values were calculated via the Mann–Whitney U test.

Beyond standard LDL-C volume reductions, the present study further elucidated the exceptional performance of the very early evolocumab intervention in improving overall “lipid quality” (Table 2, Figure 3). The evolocumab group achieved a profound, targeted clearance of the highly atherogenic small dense LDL (sdLDL) particles. Within 4 weeks, the Statin + Evolocumab group achieved a striking median sdLDL reduction rate of 69.08% [absolute decrease: 211.95 (161.32–285.82) mg/L], significantly outperforming the Statin + Ezetimibe group (48.07%; Statin + Evolocumab vs. Statin + Ezetimibe, *P* < 0.001). In turn, Statin + Ezetimibe was significantly more effective than Statin Monotherapy (30.60%; Statin + Ezetimibe vs. Statin Monotherapy, *P* < 0.001). Additionally, the evolocumab group demonstrated highly consistent superiority in lowering total cholesterol (TC) (44.31% reduction vs. 31.65%, Statin + Evolocumab vs. Statin + Ezetimibe; *P* < 0.001), and the statin plus ezetimibe group was also significantly more effective than statin monotherapy (31.65% vs. 19.53%, Statin + Ezetimibe vs. Statin Monotherapy; *P* < 0.001). These data explicitly indicate that ultra-early intensive intervention not only condenses overall lipid volume but also achieves a profound leap in lipid quality by aggressively eliminating highly pathogenic atherogenic particles.

### Subgroup analysis

**Figure 4:**
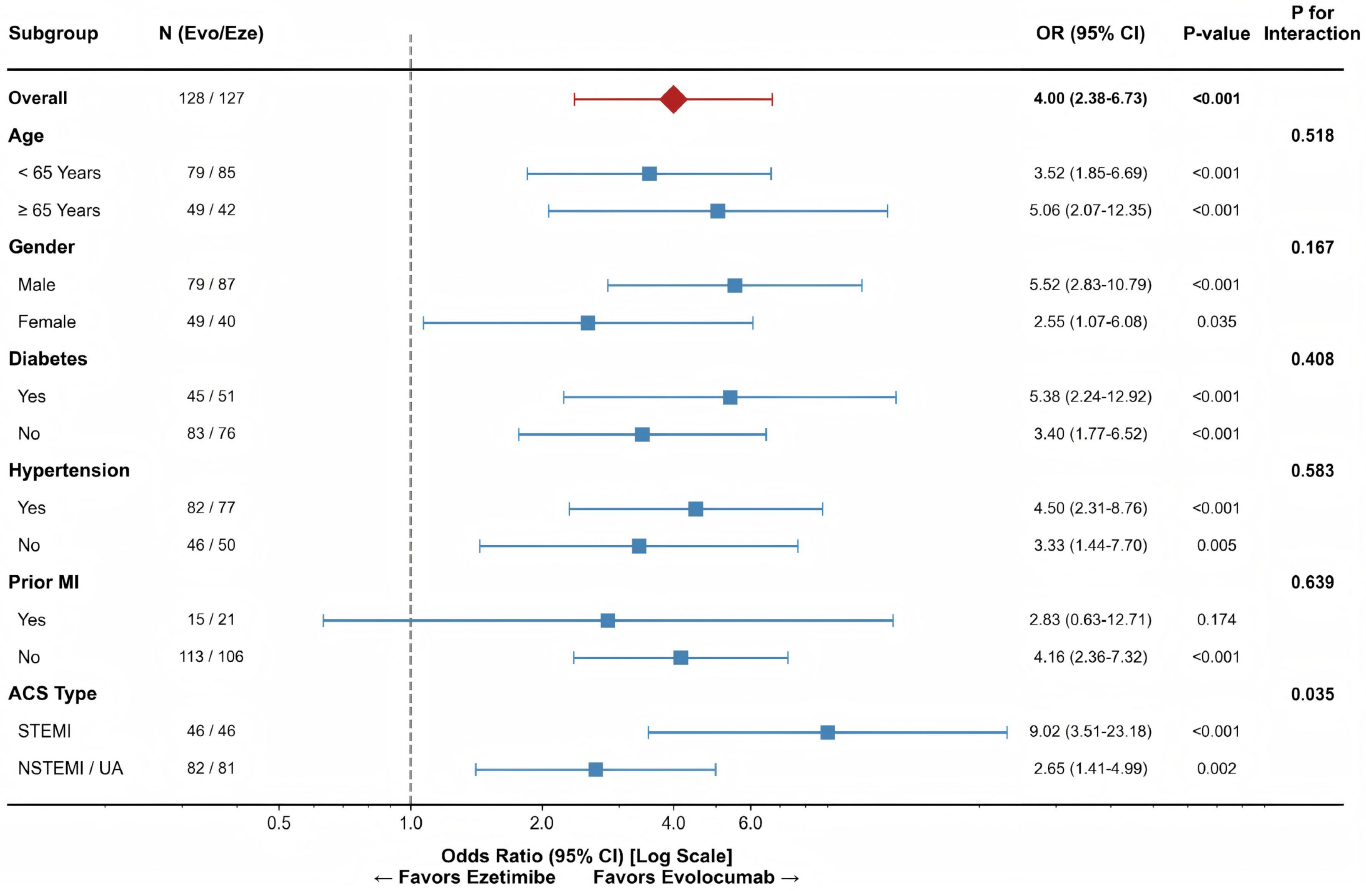
Subgroup analysis for the primary efficacy endpoint (dual LDL,C target achievement). The forest plot demonstrates the therapeutic superiority of the ultra-early Statin + Evolocumab strategy over the Statin + Ezetimibe combination across pre-specified clinical subgroups. Point estimates consistently favor the evolocumab arm, and horizontal lines indicate 95% confidence intervals (CIs). Interaction P-values (P for Interaction) are provided on the right, highlighting a remarkably consistent benefit across most strata, with a notably amplified relative benefit observed in the STEMI subgroup (P for interaction = 0.035).

To verify the broad applicability of the in-cath lab ultra-early evolocumab intervention, the primary endpoint of dual target achievement was further evaluated across multiple pre-specified clinical subgroups (Figure 4). The pronounced lipid-lowering benefit of evolocumab was remarkably consistent regardless of age, gender, baseline body mass index (BMI), or the presence of major cardiometabolic comorbidities such as hypertension or diabetes mellitus (all P for interaction >0.05). Notably, while the prior myocardial infarction (MI) subgroup did not reach intra-group statistical significance likely due to the limited sample size, the interaction analysis confirmed that the treatment effect did not differ significantly based on MI history (P for interaction = 0.639).

Furthermore, a highly compelling and significant interaction was observed concerning the ACS presentation type (P for interaction = 0.035). While the ultra-early evolocumab strategy was effective across the entire ACS spectrum, patients presenting with STEMI derived a profoundly greater relative benefit in achieving the dual target (OR 9.02, 95% CI 3.51–23.18) compared to those with NSTEMI or UA (OR 2.65, 95% CI 1.41–4.99). These data robustly validate that this intervention yields a highly consistent benefit across diverse high-risk populations, with an exceptionally amplified efficacy in the highest-risk STEMI cohort.

### Safety outcomes

The ultra-early and intensive lipid-lowering strategy was generally well-tolerated, with no unexpected safety signals identified during the 4-week follow-up period (Table 3). The overall incidence of any adverse event (AE) was comparable among the three groups: 11.8% in the statin monotherapy group, 18.1% in the statin plus ezetimibe group, and 20.3% in the statin plus evolocumab group (overall *P* = 0.171; Evolocumab vs. Ezetimibe *P* = 0.751).

**Table 3:** Safety outcomes and adverse events during the 4-week follow-up period.

Adverse Event	Statin Monotherapy (*n* = 127)	Statin + Ezetimibe (*n* = 127)	Statin + Evolocumab (*n* = 128)	Overall P-value	P-value (Evo vs. Eze)
Any adverse event	15 (11.8%)	23 (18.1%)	26 (20.3%)	0.171	0.751
ALT or AST > 3× ULN	4 (3.1%)	2 (1.6%)	2 (1.6%)	0.596	1.000
CK > 5× ULN	0 (0.0%)	0 (0.0%)	1 (0.8%)	0.370	1.000
Myalgia/Muscle weakness	4 (3.1%)	7 (5.5%)	7 (5.5%)	0.596	1.000
Gastrointestinal events	6 (4.7%)	11 (8.7%)	6 (4.7%)	0.310	0.221
New-onset diabetes	2 (1.6%)	3 (2.4%)	4 (3.1%)	0.717	1.000
Neurocognitive events	0 (0.0%)	0 (0.0%)	0 (0.0%)	NA	NA
Injection-site reactions	0 (0.0%)	0 (0.0%)	7 (5.5%)	0.001	0.014
Discontinuation due to AEs	3 (2.4%)	5 (3.9%)	8 (6.2%)	0.297	0.571

Data are presented as number (percentage). The Overall P-value reflects the comparison among the three groups using the Chi-square test or Fisher's exact test, where appropriate. The P-value (Evo vs. Eze) denotes the specific pairwise comparison between the Statin + Evolocumab arm and the Statin + Ezetimibe arm.

ALT, alanine aminotransferase; AST, aspartate aminotransferase; ULN, upper limit of normal; CK, creatine kinase; AEs, adverse events; NA, not applicable.

Crucially, the incidences of severe hepatic and muscular adverse events—major concerns in acute intensive lipid-lowering therapy—remained remarkably low and balanced across the cohorts. Elevations of ALT or AST > 3× the upper limit of normal (ULN) occurred in only 1.6% of patients in both the evolocumab and ezetimibe groups (*P* = 1.000). Similarly, clinically significant creatine kinase (CK) elevation (>5  ×  ULN) was exceptionally rare (0.8% in the evolocumab group). As expected for a subcutaneous therapy, injection-site reactions were significantly more frequent in the evolocumab arm (5.5%) compared to the oral therapy arms (0.0%) (*P* = 0.014). Furthermore, the rate of treatment discontinuation due to adverse events was low and did not differ significantly across the three groups (2.4% vs. 3.9% vs. 6.2%, *P* = 0.297), reassuring the clinical safety and tolerability of combining evolocumab with statin therapy in the acute phase of ACS.

## Discussion

In this real-world, propensity score-matched cohort study, we demonstrated that the ultra-early, in-cath lab administration of evolocumab is both highly effective and well-tolerated in patients presenting with acute coronary syndrome (ACS). The principal findings of our investigation are twofold. First, this strategy drove rapid and large-scale lipid target attainment, with 66.4% of patients achieving the rigorously defined dual LDL-C target (<1.4 mmol/L and ≥50% reduction from baseline) within merely 4 weeks (24). Second, and arguably more importantly, evolocumab exhibited an absolute advantage in the clearance of highly atherogenic small dense LDL (sdLDL) particles, achieving a substantial 69.08% median reduction (25). These results collectively underscore that ultra-early PCSK9 inhibition delivers not only an aggressive quantitative reduction in the lipid burden but also a profound qualitative leap in the lipid profile.

The imperative for rapid LDL-C lowering in the acute phase of ACS is driven by the exceptionally high risk of recurrent ischemic events associated with early plaque instability (26). While previous landmark trials, such as EVOPACS (9) and PACMAN-AMI (10), have established the efficacy in improving LDL-C levels and plaque features of initiating PCSK9 inhibitors during the index hospitalization, our study advances the intervention window to the absolute earliest clinical contact: the catheterization laboratory. Traditional oral lipid-lowering regimens, including statins combined with ezetimibe, typically require several weeks to reach their maximal pharmacodynamic effect (27), thereby leaving patients inadvertently exposed during the most vulnerable post-infarction inflammatory window (28). Our expanded three-tier comparative analysis strongly reinforces this concept. While conventional dual oral therapy (statin plus ezetimibe) marks a significant improvement over traditional statin monotherapy—validating standard guideline practices—it still leaves nearly two-thirds (66.9%) of ACS patients outside the dual-target safety window at 4 weeks. This stark contrast underscores that the ‘temporal treatment gap’ cannot be sufficiently bridged by oral escalations alone. Our data reveal that the “zero-delay” in-cath lab administration of evolocumab effectively bridges this temporal therapeutic gap. By doubling the strict dual-target achievement rate (66.4% vs. 33.1%) within a short 4-week timeframe, our ‘zero-delay’ in-cath lab strategy equips clinicians with a highly effective intervention to rapidly defuse the acute lipid-driven inflammatory cascade during the most vulnerable post-infarction window. Furthermore, our subgroup analysis revealed a novel and highly compelling interaction regarding the ACS presentation type (P for interaction = 0.035). Although the ultra-early strategy was effective universally, patients with STEMI exhibited a nearly four-fold greater relative magnitude of benefit compared to those with NSTEMI/UA. This finding implies that the intense inflammatory storm, extensive thrombotic burden, and severe plaque instability inherent to STEMI (29, 30) may render these highest-risk patients particularly responsive to the immediate and profound improvement in the circulating lipid profile driven by in-cath lab PCSK9 inhibition. This finding provides a vital mechanistic rationale for prioritizing “zero-delay” lipid interventions in patients with the most acute plaque ruptures.

Beyond the sheer reduction of standard LDL-C, the profound clearance of sdLDL observed in our cohort offers crucial mechanistic insights into the profound atheroprotective effects and lipid quality improvement of ultra-early PCSK9 inhibition. Due to their diminished size, reduced affinity for the LDL receptor, and prolonged circulation time, sdLDL particles are notoriously atherogenic (31). They readily penetrate the arterial intima, where they exhibit extreme susceptibility to oxidation, ultimately triggering intense macrophage engulfment and foam cell formation—the fundamental hallmarks of plaque vulnerability, which ultimately precipitate plaque rupture (32, 33). Our findings indicate that while traditional therapies offer only modest clearance of these pathogenic particles, the potent upregulation of hepatic LDL receptors induced by evolocumab results in a deep, targeted extraction of sdLDL (69.08% median reduction) (34, 35). This phenomenon strongly suggests that the ultra-early use of evolocumab confers a critical dual benefit: rapidly condensing the overall circulating atherogenic lipid load while fundamentally improving ‘lipid quality’ by eliminating its most inflammatory and oxidative components.

The safety profile observed in our study further substantiates the clinical feasibility of initiating an intensive lipid-lowering strategy at the ultra-early acute phase. Due to the high-pressure environment of emergency resuscitation and the acute physiological stress of the patient, clinicians are often concerned about the potential hepatic and muscular toxicities associated with the immediate administration of potent lipid-lowering agents (36). However, our results demonstrate that the ultra-early addition of evolocumab did not increase the overall incidence of adverse events. Critically, severe elevations in hepatic or muscle enzymes (e.g., ALT or AST >3× ULN, or CK > 5× ULN) were exceptionally rare in the evolocumab group and did not differ significantly from the control arms (37). Although mild local injection-site reactions were inherently more frequent with the subcutaneous administration of evolocumab, they were generally self-limiting and did not lead to a higher rate of treatment discontinuation. Furthermore, seamlessly integrating the administration timing into the routine workflow of the catheterization laboratory proved straightforward, demonstrating that this strategy is not only pharmacologically safe but also highly practical and well-tolerated in a real-world, high-acuity emergency setting.

The clinical implications of our ultra-early intervention strategy become particularly profound when contextualized within recent landmark trials. Consistent with the EPIC-STEMI study (38), our safety data reinforce that the very-early initiation of PCSK9 inhibitors in the acute setting is highly feasible and well-tolerated. However, a critical clinical question remains: does ‘zero-delay’ in-cath lab initiation translate to superior long-term outcomes compared to administration delayed by a few days post-admission? As demonstrated by the FITTER trial (39), translating lipid reductions into incremental, morphologically visible plaque regression typically requires a longer temporal window of at least several months. While our short-term data do not capture these structural changes, they demonstrate an immediate, profound condensation of the circulating atherogenic lipid load. Crucially, the FOURIER-OLE trial (40) provides compelling evidence for a ‘legacy effect,’ indicating that the earlier intensive lipid-lowering is achieved, the disproportionately greater the long-term cardiovascular benefits. By advancing the treatment window directly into the catheterization laboratory, our ‘zero-delay’ strategy immediately neutralizes the acute lipid-driven inflammatory cascade. This rapid clearance theoretically maximizes the legacy effect, establishing the earliest possible foundational milieu for subsequent plaque stabilization and long-term survival.

Despite providing valuable real-world evidence, several limitations of this study must be acknowledged. First, this study was designed as a prospective observational cohort rather than a randomized controlled trial (RCT). While RCTs remain the indisputable gold standard for efficacy evaluation, real-world observational studies offer crucial, highly practical complementary evidence that closely reflects everyday clinical practice outside of tightly controlled experimental environments. Nonetheless, despite our rigorous use of propensity score-matching (PSM) to maximally balance baseline characteristics, the lack of true randomization means that the potential impact of unmeasured residual confounding on clinical outcomes cannot be entirely ruled out (41). Future large-scale, multicenter RCTs, which our team is actively planning, are warranted to further corroborate these findings. Second, the follow-up duration of this study was relatively short (4 weeks), primarily focusing on evaluating the short-term dynamic changes in the lipid profile and the clearance rate of core pathogenic particles. There is currently a lack of continuous tracking for long-term major adverse cardiovascular events (MACE, such as cardiovascular death, non-fatal myocardial infarction, or target vessel revascularization) to confirm whether the short-term improvement in lipid quality translates into long-term survival benefits (42). Finally, this was a single-center cohort; although the final analysis reached a robust scale of 382 patients, its generalizability to broader and more diverse populations should be interpreted with caution. Future large-scale, multicenter randomized controlled trials (RCTs) are urgently needed to further corroborate the long-term clinical benefits of ultra-early, in-cath lab PCSK9 inhibitor administration (43).

## Conclusion

In conclusion, the ultra-early, in-cath lab administration of evolocumab in patients with acute coronary syndrome (ACS) drives a more rapid and large-scale achievement of stringent dual LDL-C targets compared to standard conventional therapies. Furthermore, it achieves a profound and targeted clearance of highly atherogenic small dense LDL (sdLDL) particles, which are a major culprit in plaque instability. This study confirms the dual superiority of very early PCSK9 inhibition in delivering both “rapid lipid lowering” and “deep lipid quality improvement” without conferring any additional safety risks. These robust real-world data strongly support the clinical practice of shifting the window of intensive lipid intervention forward to the catheterization laboratory for high-risk ACS populations.

## Data Availability

The original contributions presented in the study are included in the article/Supplementary Material, further inquiries can be directed to the corresponding author/s.
